# Adolescent condom negotiation: a concept analysis and conceptual framework

**DOI:** 10.3389/frph.2026.1764331

**Published:** 2026-01-29

**Authors:** Mary A. Antwi, Jacob D. Dogtir, Khairul A. M. Islam, Danielle C. Alcena-Stiner

**Affiliations:** 1 School of Nursing, University of Rochester, Rochester, NY, United States; 2 Center for Interdisciplinary Research on AIDS, Yale University, New Haven, CT, United States

**Keywords:** adolescent condom negotiation, communication, concept analysis, negotiation skills, nursing research, sexual health

## Abstract

**Introduction:**

Adolescents are at risk of sexually transmitted infections and unintended pregnancies. Consistent condom use is effective in curbing the double burden of diseases and unplanned conceptions. Condom negotiation influences consistent condom use. However, a clear operational definition is lacking. This paper analyzes the concept of adolescent condom negotiation and examines its defining attributes, antecedents, and consequences to promote conceptual clarity and enhance sexual health research and practice.

**Methods:**

Norris's (1982) concept clarification method guided this analysis. This framework systematically observes, describes, and organizes conceptual evidence to support the development of an operational definition and a preliminary conceptual model of adolescent condom negotiation. A comprehensive literature review was conducted using PubMed, CINAHL, and PsycINFO, as well as the reference lists of relevant studies. Studies were included if they addressed communication, negotiation, or decision-making related to condom use among adolescents.

**Results:**

Twenty-seven sources met the inclusion criteria. Two core attributes of adolescent condom negotiation were identified: (1) communication strategies used to express preferences regarding condom use, and (2) negotiation behaviors aimed at reaching mutual agreement with a partner. The analysis resulted in an operational definition and a preliminary conceptual model that illustrates how communication and negotiation processes influence condom use among adolescents. No existing instrument designed to specifically measure adolescent condom negotiation was identified.

**Discussion:**

The findings of this study will help researchers, nurses, doctors, and sexual health educators design more precise measurements and focused interventions for teenagers, especially in environments with high STI and teenage pregnancy burdens and gendered power inequalities. Operationalizing adolescent condom negotiation marks a critical step toward advancing adolescent sexual health research and practice. A clear, evidence-based definition supports targeted education and interventions to promote consistent condom use among adolescents, thereby reducing the risks of sexually transmitted infections STIs and unintended pregnancies.

## 1 Introduction

Adolescence is characterized as the second decade of life, with approximately 1.2 billion adolescents globally, accounting for 16% of the world's population (1). Adolescents undergo significant developmental changes across physical, psychological, and social domains (2). Adolescence also marks a critical developmental phase for sexual maturation, necessitating thorough education to effectively manage their evolving circumstances. Adolescence is also a crucial time for the development of health literacy (3). Their ability to access, appraise, and utilize health information can impact their sexual health outcomes through making informed decisions, hence the need for parents, teachers, and healthcare providers to start age-appropriate sexual health discussions with adolescents early (4).

Adolescents are seen as a window of hope to fuel a tangible improvement in sexual and reproductive health due to their inclination to try out new habits, thus sparking innovation and progress in safe sex behaviors (1). Unfortunately, many adolescents lack adequate comprehensive sexual and reproductive health information to help them make the right decisions regarding their sexual relationships (5). In many developing countries, particularly in Sub-Saharan Africa, adolescent sexuality largely remains a taboo topic (6). Yet, sexual initiation in adolescence is prevalent (2). Initiating sexual activity without adequate knowledge and skills in safe sex practices exposes this young and vulnerable population to a higher risk of negative sexual health outcomes, such as unintended pregnancies that often end in unsafe abortions, and STIs, including Human Immunodeficiency Virus (HIV) infection (1).

In the United States, while the prevalence of sexually risky activities and experiences among teenagers declined between 2009 and 2019, the incidence of STIs between 2014 and 2018 saw an increase in the adolescent population (7). Additionally, in 2020, adolescents and young adults accounted for more than half of all STIs in the United States (8). These findings show that promoting safe sex among teenagers is critical in reducing the global rates of STIs. Additionally, the disconnect between the decrease in risky sexual practices and increasing STI rates among adolescents underscores the critical need for operational definitions and reliable measurement of adolescent sexual health concepts such as sexually risky behavior, safe sex, and condom use and negotiation. In fact, researchers must revisit how adolescents define sex, because studies indicate that adolescents focus on penile-vaginal sex when asked about risky sex (9). Young people tend to ignore other aspects of risky sexual behavior, such as unprotected oral sex, which also carries risks of orally transmitted infections such as chlamydia, gonorrhea, syphilis, and human papillomavirus (HPV) infection, which can cause oropharyngeal squamous cell carcinoma (10). Dental dams present a simple and yet effective physical barrier for safe oral sex, but unfortunately, they are underutilized (9). There is also limited education on their use and a paucity of data on the use of dental dams, especially among adolescents, as the available research has focused solely on adult women (10).

Teenage pregnancy is a public health problem that leads to severe negative outcomes among adolescent girls and their neonates. An estimated 21 million adolescent girls from developing countries got pregnant in 2019 (11), with 50% being unintended. In the US, although the rate of adolescent pregnancy is seen to be low when compared with low- and middle-income countries, it is still the highest among high-income countries (12). In low and middle-income countries, about 55% of unplanned conceptions among teenagers end up in abortions, mostly under unsafe hygienic conditions or conducted by unskilled personnel (11). Furthermore, adolescent moms (ages 10–19) are more likely than women (ages 20–24) to experience eclampsia, puerperal endometritis, and systemic infections (1). Adolescent mothers' neonates are also more likely to experience low birth weight, premature delivery, and serious neonatal conditions (1). Pregnancy-related complications remain the highest cause of morbidity and mortality among adolescent girls in developing countries (1). Therefore, achieving the Sustainable Development Goals pertaining to maternal and neonatal health largely rests on preventing teenage pregnancy and empowering adolescents to avoid risky sexual practices (1).

Consistent correct use of condoms is an effective way to reduce the double burden of STIs, including HIV, and unintended pregnancies (13). The prevalence of HIV has decreased throughout sub-Saharan Africa due to increased condom use (14). The literature on condom use among adolescents in the context of HIV and other STI prevention has often highlighted the barriers to condom availability among teenagers (15). However, other studies have identified that even with increasing availability, access remains a barrier to adolescents, citing issues such as socio-cultural. Thus, in recent research, attention has been drawn to other enabling factors that could promote condom use among adolescents (16).

Negotiation is a concept that varies in meaning across different contexts (17, 18). Moreover, the literature indicates that adolescent condom negotiation has been measured using various scales (19, 20) and is referred to by different terms in various texts, such as safer sex negotiation (21). This highlights the need for a precise definition of the concept.

To generate nursing knowledge, it is essential to identify and clarify concepts pertinent to diverse populations and contexts (22). The clarified elements must then be incorporated into research questions or hypotheses, which will be empirically tested to provide evidence that contributes to the advancement of nursing science (23). An analysis of adolescent condom negotiation will elucidate this phenomenon through meaningful description (23). The clarification of the concept of adolescent condom negotiation is essential for understanding the variations in terminology and measurement across different contexts and studies. An operational definition of the concept can be utilized by nurses working with adolescent populations to improve consistency in identifying adolescent concept negotiation in practice. The components of the concept can be utilized to define variables for subsequent nursing research. This clarification of concepts could provide foundational support or enhance a theory aimed at advancing condom negotiation, an area currently underrepresented in the literature.

## 2 Methods

The primary purpose of this analysis is to establish an operational definition of adolescent condom negotiation. This refers to the specific, measurable, and observable behaviors and communication strategies that adolescents use to achieve or avoid condom use with a sexual partner. As a “set of behaviors, not a singular act”, it involves both verbal and non-verbal communication. Additionally, a conceptual framework was developed and subsequently used to generate hypotheses related to adolescent condom negotiation. These are valuable for recognizing the concept in practice and guiding subsequent research inquiries based on the hypotheses formulated during this analysis.

### 2.1 Article type: conceptual analysis and review

Conceptual analysis is a systematic process that aims to break down a term, clarifying the critical attributes that distinguish it from comparable terms. The outcome is a collection of defining traits that enable a researcher to establish a precise definition of the concept, select suitable and effective measurement instruments, or ascertain the necessity for new tools, and recognize the concept in practical applications or research data (24). Various methods are utilized in concept analysis, including those proposed by Walker and Avant (25), Rodgers and Knafl (26), and Norris (23). This analysis employs the methodology established by Norris (23), which delineates five steps as follows:

After identifying the concept of interest, observe and describe the phenomenon repeatedly, and if possible, describe the phenomenon from the point of view of other disciplines.
Systematize the observations and descriptions.
Derive an operational definition of the concept under study.
Produce a model of the concept that includes all its component parts.
Formulate hypotheses (22, 23).

### 2.2 Data sources

The first author conducted the literature search for this analysis in the PubMed, CINAHL, and PsycINFO databases. Assistance was obtained from the medical librarian at the University of Rochester's School of Nursing to identify and finalize a comprehensive set of search terms. The key terms and phrases utilized in this search were adolescent, adolescence, teenager, teen, “sexual negotiation”, “condom negotiation”, or “condom negotiated” or “condom negotiates” or “condom negotiating”. These terms were used in various combinations to conduct a comprehensive search. The final search strategies are outlined in Table 1. From CINAHL, 104 sources were identified, 57 from PsycINFO and 102 from PubMed. The following inclusion criteria were applied in the literature search: peer-reviewed literature written in the English language and published between 1994 and 2025, specifically focusing on human subjects within the adolescent age range of 10 to 19 years old. The literature search publication timeline was set for the year 1994, as that year marked the first time adolescent sexual and reproductive health gained global attention (27). All searches yielded a total of 263 sources. One article (28) was frequently cited by other articles and was the first to give a clear definition of adolescent condom negotiation. Once the search was concluded, all sources were further evaluated using reference management software (EndNote, Clarivate) for a more comprehensive eligibility assessment. Among the 263, 102 (39%) were duplicates and were removed. The remaining 161 literature sources' titles and abstracts were screened in the next step, out of which 58 (36%) sources had no information on condom negotiation and were removed. Full texts of the remaining 103 sources were then retrieved for screening. Further eligibility screening led to the exclusion of 76 (74%) sources. These were excluded because they focused on older people or had a small representation of youth under 19 years (*n* = 71, 93%) and languages other than English (*n* = 5, 7%). The final literature source count was *n* = 27.

**Table 1:** Search strategy in databases.

The search on PubMed utilized the following search strategy: (“condom negotiation"[All Fields] AND (“adolescences"[All Fields] OR “adolescency"[All Fields] OR “adolescent"[MeSH Terms] OR “adolescent"[All Fields] OR “adolescence"[All Fields] OR “adolescents"[All Fields] OR “adolescent s"[All Fields] OR “teen*"[All Fields])) AND ((excludepreprints[Filter]) AND (humans[Filter]) AND (english[Filter]) AND (adolescent[Filter])).
The search strategy on CINAHL and PsycINFO:
The search strategy (adolescence or adolescent or teenager or teen) AND (sexual negotiation or condom negotiation or “condom negotiated” or “condom negotiates” or condom negotiating) was applied in CINAHL and PsycINFO because these databases do not utilize MeSH terms and other edits such as [All Fields] and [Filter] found in PubMed.

The concept of adolescent condom negotiation was identified across all texts; thereafter, data were extracted regarding its description, measurement, or application. The characteristics of the concept were extracted and structured into a raw data file of antecedents and outcomes (Table 2) and attributes (Table 3). The data were analyzed and reorganized to identify the primary attributes, antecedents, and outcomes of adolescent condom negotiation (Table 4).

**Table 2:** Adolescent condom negotiation, preliminary raw data extraction, antecedents and consequences.

Literature source	Antecedents	Literature source	Consequences	Notes
(49)	Confidence in talking about sexual health issues with a partner	(41)	Consistent condom use	Sexual coercion negatively impacts condom negotiation
(44)	Self-efficacy in condom negotiation	(44)		Psychological effects of abuse and alcohol intoxication negatively impact condom negotiation
(31)	Sexual self-efficacy	(31)	Negative and positive outcomes, fear of relationship ending when negotiating condom use.	Fear of condom negotiation related to depression outcomes
		(19)	Higher condom use	
		(40)	Fear of condom negotiation is associated with inconsistent condom use. High condom negotiation is associated with high HIV risk perception	

**Table 3:** Adolescent condom negotiation attributes.

		Definition Parts			
Number	Source	Communication	Partners	Outcome	Identified patterns
1	(28)	✓	✓	✓	Gender differences Various strategies
2	(45)	✓	✓	✓	
3	(49)	✓	✓	✓	Face to face interactions, Communication through sexting
4	(31)	✓	✓	✓	
5	(32)				Gender differences, girls had higher condom negotiation compared to boys
6	(39)	✓	✓	✓	Condom negotiation efficacy (CNE) Teachers’ role is significant in developing CNE
7	(20)	✓	✓	✓	Gender differences
8	(21)	✓	✓	✓	Safer sex negotiation included refusing partner sex
9	(50)	✓	✓	✓	Partner negotiation for condom use, Ability to convince
10	(19)	✓	✓	✓	Condom negotiation frequency is negatively impacted by alcohol and other drug use
11	(43)	✓	✓	✓	Power relations affect condom negotiation
12	(44)	✓	✓	✓	Physical abuse affects adolescent condom negotiation among girls who are victims
13	(42)	✓	✓	✓	Gendered roles and power relations influence condom negotiation; FGM negatively impacts condom negotiation
14	(47)	✓	✓	✓	
15	(48)	✓	✓	✓	
16	(33)	✓	✓	✓	Safer sex negotiation including refusing sex and requesting condom use
17	(51)	✓	✓	✓	
18	(46)	✓	✓	✓	Safer sex negotiation
19	(14)	✓	✓	✓	Does not clearly define the concept; Gender differences with women finding it more difficult to negotiate for condom use
20	(36)	✓	✓	✓	Both boys and girls agree it is more difficult for girls to negotiate condom use
21	(34)	✓	✓	✓	Various strategies including withholding sex, directly asking for condom use, using seduction or deception, using risk information and relationship conceptualization
22	(41)	✓	✓	✓	Attitudes towards condom negotiation measured
23	(40)				Did not give a direct definition but measured fear of the concept
24	(38)				Did not give a direct definition but measured fear of the concept; Parental communication with adolescents reduced the fear of condom negotiation except in authoritarian parental approaches
25	(37)				Does not give a clear definition of the concept Parental communication can promote concept
26	(52)				Does not give a definition but measures negotiation efficacy Higher gender equity perception led to higher condom negotiation
27	(35)	✓	✓	✓	Age disparities in adolescent sexual relationships affect condom negotiation. Various strategies. Use of oral contraceptives prevents acceptance of condom use

**Table 4:** Reorganized condom negotiation attributes, antecedents, and outcomes.

Condom negotiation attributes	Descriptions	Literature sources
Communication	Discussions, making requests verbally or non-verbally	(14, 19–21, 28, 31, 33–43, 45–52)
Skill	Employing various strategies to get a partner to use condoms	(14, 33, 39, 46, 51, 2022, 14, 19–21, 34, 36, 38, 40, 41, 43, 50)
Condom negotiation antecedents	Descriptions	Sources
Adolescent sexual partners	Young sexual partners between the ages of 15 and 19 years	(14, 19–21, 28, 31, 33–43, 45–52)
Sexual Self-efficacy	Confidence in the ability to request condom use	(14, 19–21, 31, 33, 34, 36, 39–43, 46–51)
Fear of unintended pregnancy and STIs	The fear of falling pregnant or contracting an STI through condomless sex	(19–21, 31, 39, 43, 49, 50)
Condom negotiation outcomes	Descriptions	Sources
Condom use	Using condoms during sexual intercourse after condom negotiation	(19, 33, 37, 38, 40–43, 45–48, 50, 51)
Non-use of condoms	Having condomless sex even after condom negotiation with a partner	(20, 21, 31, 34–36, 39, 49)

## 3 Results

### 3.1 Defining the concept (step 1)

#### 3.1.1 Uses other than those specific to adolescent condom negotiation

Negotiation is characterized by the Oxford Dictionary as a formal discussion aimed at reaching an agreement (29) whereas the Cambridge Dictionary describes it as the process of engaging in discussions to achieve an agreement (29). The definitions illustrate a reciprocal dialogue among parties aimed at achieving consensus, while the Oxford dictionary adopts a more formal tone.

Various applications of the term “negotiation” were observed across distinct disciplines. Negotiation is characterized as a formal process in international trade and conflict resolution (17, 18). In the field of law, negotiation constitutes an official responsibility of a lawyer (30). These disciplines advocate for a structured, decision-focused approach to negotiation. These disciplines also differ significantly from negotiating in an intimate context. Secondly, in the fields of law and international trade, the involvement of a third-party negotiator is typically observed. In these disciplines, negotiation often involves third-party participation to provide an impartial perspective, facilitating the swift and effective resolution of disputes (18). Timing is critical, as negotiations across various disciplines often take place amid ongoing trade or conflicts.

#### 3.1.2 Uses specific to adolescent condom negotiation

The concept of condom negotiation in nursing was first introduced in published literature in 1989 by Weisman and colleagues, who examined adolescents’ perceived AIDS risk and preventive behaviors, defining it as the act of requesting condom use from a partner. The nursing literature typically categorizes adolescent condom negotiation as informal behavior (31, 32). Condom negotiation, as outlined in the referenced sources, is predominantly examined within the framework of two sexual partners (20, 33), with no reference to a third party. The timing of negotiation is also characterized in the literature as an activity that occurs during a sexual encounter, rather than before or after (34, 35). Several articles discussed various strategies that adolescents employed in condom negotiation (28, 34). These strategies encompassed various forms of deception, such as claiming to have an infection or the potential for pregnancy (despite being on birth control), thereby necessitating the use of condoms (34). In addition to verbal methods of condom negotiation, non-verbal strategies exist, such as employing seduction by producing a condom and offering it to the partner during heightened arousal (28).

#### 3.1.3 Attributes-communication and skill

Further patterns identified in the studies encompassed characterizations of negotiation related to communication, self-efficacy, and a skill that adolescents can acquire and refine through practice. Condom negotiation is characterized in various studies as a communicative skill that adolescents can acquire (28, 36, 37). Adolescent condom negotiation is characterized as a complex process that extends beyond individual-level partner persuasion, involving influences from teachers, parents, and peers in the development of this skill (37–40). The previously referenced articles that examined the associations between adolescent sexual discussions with adults indicate that sexual health communication among adolescents, parents, and teachers is significantly linked to the development of condom negotiation skills. Research indicates a consistent perspective across multiple studies (14, 35, 41) that gender differences exist in condom negotiation between female and male adolescents. Gendered patterns suggest that males view condom negotiation as a feminine responsibility, yet the ultimate decision to use condoms rests with men (42). Power imbalances in adolescent condom negotiation are closely linked to gendered norms (43) and (35) identified that adolescents who engaged in sexual relations with older partners or those involving monetary favors exhibited low levels of condom negotiation. Other studies identified the adverse effects of drug and alcohol use prior to sexual encounters on condom negotiation (19, 44, 45). Condom negotiation was infrequent among adolescents who engaged in drug and alcohol use. Research indicates that condom negotiation varies according to the type of relationship (41). Sexual intercourse with casual partners, particularly in contemporary “friends with benefits” arrangements, typically involves greater negotiation regarding condom use compared to sexual encounters with long-term partners (41).

#### 3.1.4 Condom negotiation measurement

The measurement of condom negotiation among adolescents varied across the reviewed studies. Most authors utilized the Measurement of Condom Attitudes Scale (MCAS) to assess condom negotiation, which primarily evaluates condom attitudes rather than negotiation itself. An exception is Singer et al. (19), who used the Sexual Communication Scale for this measurement. Additional studies utilized the MCAS to assess condom negotiation, encompassing discussions about condom use and complete refusal of sex, referred to as safer sex negotiation (21, 33, 46). Some articles did not provide a clear definition of adolescent condom negotiation, despite measuring this outcome (38, 40, 47, 48). However, these articles contained implicit assumptions regarding the concept based on the measurement methods employed, which were extracted as part of the data for this paper. Identifying alternative measures would be beneficial.

#### 3.1.5 Antecedents and outcomes

Multiple studies indicate that sexual self-efficacy served as a precursor to adolescent condom negotiation (19, 42–44, 49, 50). This pertains to the confidence that adolescents exhibited in discussions regarding condom use (39, 50). Research indicates that discussions regarding safe sex practices between parents (37, 38) and teachers (39) significantly contribute to adolescents' development of sexual self-efficacy, subsequently facilitating condom negotiation. Adolescent girls who have experienced any form of intimate partner abuse or coercion exhibit notably low levels of sexual self-efficacy (41, 43). The motivation to prevent STIs and unintended pregnancies contributed to adolescent condom negotiations (31, 39, 49). The authors recognized this risk behavior as an inconsistent precursor to condom negotiation, as it infrequently led to such negotiations (19, 20). The primary outcome of condom negotiation was reported to be consistent condom use (19, 49). Condom use was frequently mentioned in the text; however, instances of non-use were also noted (31). However, as previously noted, increased condom use was frequently observed as a result.

### 3.2 Systematizing the observations and descriptions (step 2)

The second step of the Norris concept analysis approach involves categorizing the distinct components of the descriptions of the phenomena (23). In this context, adolescent condom negotiation can be defined as the communicative skill employed by adolescent sexual partners, predicated on sexual self-efficacy, resulting in either the utilization or non-utilization of condoms. These observations mapped out the antecedents, attributes, and outcomes of adolescent condom negotiation. Multiple papers delineated the process of teenage condom negotiation as a protective competency aimed at preventing the acquisition of STIs or unintended pregnancies through condom use (19, 20, 43, 51). Articles addressing this primary objective of assessing condom negotiation (21, 33, 46) aimed to determine its impact on the consistent use of condoms during sexual intercourse among adolescents. Interestingly, unintended pregnancies and STI susceptibility less frequently prompted adolescents to negotiate for the use of condoms (21, 31, 49). All sources characterized teenage condom negotiation as a process of communication. This communication is recognized as a multifaceted activity that employs several strategies. These strategies encompass both verbal and non-verbal methods (28, 34, 38, 40). While communication generally indicated an acceptance of condom usage (32, 49), other authors noted that condom negotiation did not consistently result in actual condom use (33, 46).

Fear of communicating the desire for the use of condoms was also reported by some texts (31, 41, 44). These studies pointed to prior mental health problems experienced by girls, such as physical abuse and sexual coercion, depression, and the use of alcohol and drugs, serving as critical limiting factors against condom negotiation. On the other hand, some sources presented factors that served to promote adolescent condom negotiation. These included high HIV risk perception (40), communication with parents about sex (37, 38), higher gender equity perception (52), and communication with teachers about sexual health issues (39). These condom negotiation promotive factors served to increase sexual self-efficacy, particularly among girls, which helped to increase their confidence to request condom use with their partners.

### 3.3 Deriving an operational definition (step 3)

Norris presents a distinct viewpoint compared to others regarding the formulation of an operational definition (53), which constitutes the third step in her analytical framework. An operational definition, as described by Norris, addresses the question of how to identify the concept in practice (22, 23). Other scholars emphasize the importance of measurement, which is absent from her approach (53). Adolescent condom negotiation is hereby defined as the proficient use of verbal and non-verbal communication strategies by adolescent sex partners to discuss condom use during sexual intercourse, aiming to reach a consensus on whether to use condoms or not. This skill is consistently preceded by the sexual self-efficacy of the partners, and less frequently by concerns regarding unintended pregnancies and sexually transmitted infections.

### 3.4 Develop a model of the concept that encompasses all its component parts (step 4)

**Figure 1:**
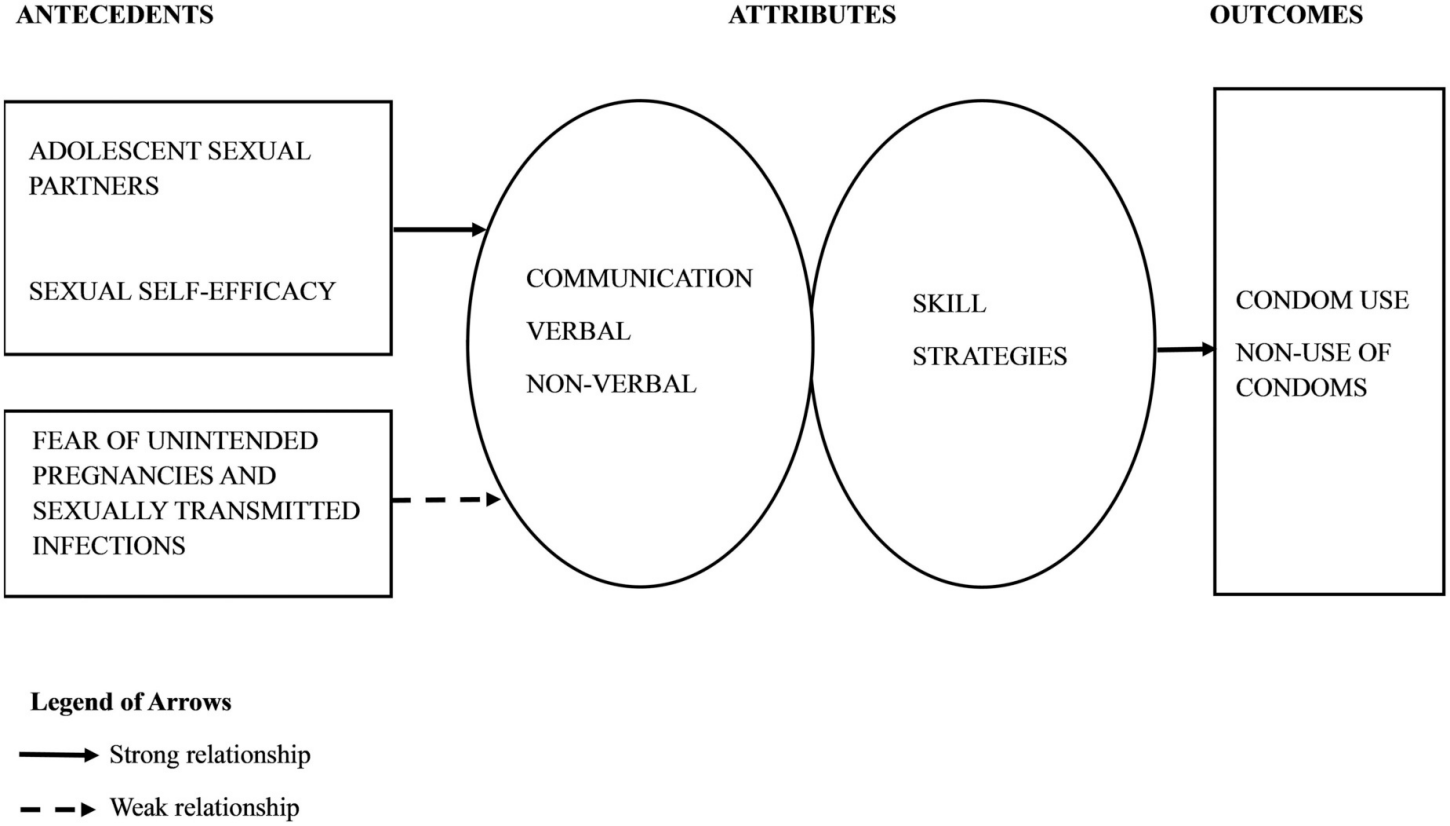
Conceptual model of adolescent condom negotiation.

Lackey (22) posits that models enhance the understanding of data by facilitating generalizations regarding concepts and clarifying the relationships among categories, patterns, and hierarchies that have been established. The outcomes of concept clarification should be communicated more effectively through a well-structured model. In the fourth step of Norris' method, a model of adolescent condom negotiation was developed, detailing the relationships among the identified categories, antecedents, and outcomes of the concept. This model functions to convey information regarding the concept (22). Figure 1 represents the conceptual model of adolescent condom negotiation derived from this concept analysis of adolescent condom negotiation.

### 3.5 Formulate hypotheses (step 5)

The final step of Norris' method involves formulating hypotheses to evaluate the concept. Lackey (22) states that the model and the resulting operational definition are utilized to formulate hypotheses. The subsequent directional hypotheses were formulated based on the model and operational definition of adolescent condom negotiation. (1). Increased sexual self-efficacy among adolescents is associated with a higher frequency of condom negotiation. (2). Sexual self-efficacy serves as a more significant predictor of condom negotiation compared to the fear of unintended pregnancy.

## 4 Discussion

### 4.1 Conceptual findings and implications for theory

The purpose of this concept analysis was to identify an operational definition for adolescent condom negotiation. This was accomplished by repeatedly describing the concept, its attributes, antecedents, and outcomes, and eventually identifying the categories and common themes that emerged across the literature sources. The purpose was met, and results revealed an operational definition of adolescent condom negotiation, which was then graphically presented in a conceptual model.

This analysis identified that the concept of adolescent condom negotiation encompasses skills of verbal and non-verbal communication, which takes place in the context of sexual intercourse between adolescent sexual partners. It is preceded by sexual self-efficacy and, less frequently, the fear of unintended conceptions and STIs. Conceptually, adolescent condom negotiation resulted in either the use or non-use of condoms.

The literature on adolescent sexual health often purports that adolescent condom negotiation always has an outcome of condom use, but this is not always true. Other texts indeed have identified that while condom use was assumed, the contrary outcome was the reality (34). Therefore, it is essential for scientists to dispel this presumption.

Condom negotiation is a process that may or may not result in condom use. Power dynamics between partners, sexual communication and assertiveness, trust, perceived danger of STIs, and gender or cultural norms affect adolescent condom negotiation. Successful condom negotiation requires the ability to express desires and overcome condom stigma or rejection. Thus, condom negotiation must be understood as a process rather than a binary outcome in research measurement and to create interventions that improve communication, sexual autonomy, and safer sexual behaviors.

Gendered patterns and power imbalances were revealed, placing the female or weaker partner at the requesting end and the male or more powerful partner at the final decision-making end. Negotiation may be conceptually inadequate in such condom use contexts of gender and power imbalances. Negotiation requires reciprocal communication and shared decision-making power, yet in relationships with unequal gender dynamics, one partner may control condom use. When the more powerful person makes the ultimate decision, the less powerful partner has little agency and may be better understood through the lens of condom decision-making dynamics, power-mediated condom decision-making, or condom use communication. This phrase effectively conveys how structural and relational power disparities affect sexual health behaviors. Reframing condom negotiation in this way highlights the need for interventions that address communication skills and sociocultural and gendered power structures that limit real shared decision-making in sexual relationships.

Closely linked to gendered power imbalances is the fact that the most commonly used barrier method in sexual intercourse is the male condom, which places more control over its use on the male partner. Indeed, even though female condoms exist, none of the studies reviewed had a focus on them. It is important to consider the protection of girls through education and the promotion of female condom use among adolescents.

Furthermore, this conceptual work identified that the nuances in adolescent sexual relations, such as oral sex performance or receiving, are not frequently captured in research within this population. This lack of research attention to other forms of sexual acts besides penile-vaginal intercourse could be linked to adolescents' perceptions of engaging in less sexually risky behavior, yet having a consistently high incidence of STIs. Sexual health research in the adolescent population should inclusively examine oral sex behavior and the promotion of safe practices, such as the use of dental dams.

Additionally, this conceptual work identified condom negotiation to be a component of safer sex practices, which included both condom use and not engaging in intercourse if agreement is not reached to use condoms. However, a question that immediately arises is, how can a sexual activity be described as safe when it did not occur at all? Therefore, the authors recommend that condom negotiation should be viewed as a distinct concept, separated from abstinence, to facilitate more accurate measurement of the concept.

Lastly, this conceptual analysis identified that the measurement scale for condom negotiation was either the MCAS or the Sexual Communication Scale. Neither scale captures the concept as revealed in this analysis. Future research needs to include specific scale development using the operational definition and model developed in this concept analysis of adolescent condom negotiation.

### 4.2 Next steps

To the best of the authors' knowledge, this concept analysis is the first one done on condom negotiation, specifically in the population of adolescents. This concept analysis of adolescent condom negotiation, therefore, contributes to the literature by providing a clear operational definition of the concept and clarifying its meaning, presenting it as a measurable phenomenon (22).

Additionally, this concept clarification could serve as background work or contribute to a theory that focuses on promoting condom negotiation, which is currently lacking in the literature. Also, more research should be conducted on how the fear of unintended conceptions and STIs contributes to adolescent condom negotiations. This is critical to establish the significance of the relationship between fear of unintended pregnancy and STIs and condom negotiation among adolescents.

Although Bergdahl and Berterö (54) argue that concept analysis should be discarded since it is not a component of a strong scientific approach, it can be counterargued that concept analysis, when done systematically and tied to the clinical problem under study, produces effective data for further research (55). Thus, this concept analysis has strictly followed a systematic and transparent process for literature search, data extraction, and analysis of findings to generate valid evidence that informs further data collection on adolescent condom negotiation and use.

### 4.3 Strengths and limitations

This analysis has some limitations. Only English-language publications were included so that authors could clearly understand the content of each article in the analysis. However, articles from journals that publish in multiple languages were included in the search, potentially reducing linguistic bias through the inclusion of English translations that met all other inclusion criteria, as was the case in the paper authored by de Melo and colleagues (20). Furthermore, because the selection, data extraction, and analysis processes were conducted by a single researcher, there may be some bias in these areas. However, this bias was minimized by strictly following systematic procedures and through further checks by co-authors. Given the preliminary nature of the concept analysis findings, expert interviews are necessary to inform the conceptualization of adolescent condom negotiation, which may change as the researchers’ comprehension of the idea matures or evolves through further research (25).

Nevertheless, this concept analysis identified a significant gap in the clarity of a long-standing concept in published texts and contributes to the literature an operational definition of adolescent condom negotiation, along with subsequent hypotheses. These can be further explored in research, both qualitative and quantitative studies, for a more in-depth understanding of adolescent condom negotiation.

### 4.4 Conclusions

This paper aimed to conceptually analyze the concept of adolescent condom negotiation using Norris' (23) approach to derive an operational definition of the concept. The goal of this work was met with systematic and transparent procedures. The results of this work can be used as a contribution to the development and testing of an adolescent condom negotiation scale. The operational definition of the concept can also be utilized by researchers in the field of adolescent health for clarity in their studies.
