# Correction: Adolescent condom negotiation: a concept analysis and conceptual framework

**DOI:** 10.3389/frph.2026.1810959

**Published:** 2026-03-06

**Authors:** Mary A. Antwi, Jacob D. Dogtir, Khairul A. M. Islam, Danielle C. Alcena-Stiner

**Affiliations:** 1 School of Nursing, University of Rochester, Rochester, NY, United States; 2 Center for Interdisciplinary Research on AIDS, Yale University, New Haven, CT, United States

**Keywords:** adolescent condom negotiation, communication, concept analysis, negotiation skills, nursing research, sexual health

In the published article, an incorrect **Funding** statement was displayed as “The author(s) declared that financial support was not received for this work and/or its publication.” The correct funding statement reads: “The author(s) declared that financial support was received for this work and/or its publication. The publication of research reported in this article was supported by National Institute on Mental Health of the National Institutes of Health under award number R25MH087217.’’

The author contributions statement was erroneously given as “MA: Conceptualization, Writing – review & editing, Formal analysis, Methodology, Writing – original draft, Data curation. JD: Writing – original draft, Writing – review & editing. KI: Writing – review & editing, Writing – original draft. DA-S: Supervision, Writing – original draft, Writing – review & editing, Conceptualization, Validation.”

The correct author contributions statement is “MA: Conceptualization, Writing – review & editing, Formal analysis, Methodology, Writing – original draft, Data curation. JD: Writing – original draft, Writing – review & editing. KI: Writing – review & editing, Writing – original draft. DA-S: Supervision, Writing – original draft, Writing – review & editing, Conceptualization, Validation, Funding acquisition.”

The original version of this article has been updated.

